# The Protein Partners of GTP Cyclohydrolase I in Rat Organs

**DOI:** 10.1371/journal.pone.0033991

**Published:** 2012-03-27

**Authors:** Jianhai Du, Ru-Jeng Teng, Matt Lawrence, Tongju Guan, Hao Xu, Ying Ge, Yang Shi

**Affiliations:** 1 Department of Surgery, Medical College of Wisconsin, Milwaukee, Wisconsin, United States of America; 2 Children's Research Institute, Medical College of Wisconsin, Milwaukee, Wisconsin, United States of America; 3 Cardiovascular Center, Medical College of Wisconsin, Milwaukee, Wisconsin, United States of America; 4 Department of Pediatrics, Medical College of Wisconsin, Milwaukee, Wisconsin, United States of America; 5 Human Proteomics Program and Department of Physiology, School of Medicine and Public Health, University of Wisconsin-Madison, Madison, Wisconsin, United States of America; 6 Patient Centered Research, Aurora Health Care, Milwaukee, Wisconsin, United States of America; Nagoya University, Japan

## Abstract

**Objective:**

GTP cyclohydrolase I (GCH1) is the rate-limiting enzyme for tetrahydrobiopterin biosynthesis and has been shown to be a promising therapeutic target in ischemic heart disease, hypertension, atherosclerosis and diabetes. The endogenous GCH1-interacting partners have not been identified. Here, we determined endogenous GCH1-interacting proteins in rat.

**Methods and Results:**

A pulldown and proteomics approach were used to identify GCH1 interacting proteins in rat liver, brain, heart and kidney. We demonstrated that GCH1 interacts with at least 17 proteins including GTP cyclohydrolase I feedback regulatory protein (GFRP) in rat liver by affinity purification followed by proteomics and validated six protein partners in liver, brain, heart and kidney by immunoblotting. GCH1 interacts with GFRP and very long-chain specific acyl-CoA dehydrogenase in the liver, tubulin beta-2A chain in the liver and brain, DnaJ homolog subfamily A member 1 and fatty aldehyde dehydrogenase in the liver, heart and kidney and eukaryotic translation initiation factor 3 subunit I (EIF3I) in all organs tested. Furthermore, GCH1 associates with mitochondrial proteins and GCH1 itself locates in mitochondria.

**Conclusion:**

GCH1 interacts with proteins in an organ dependant manner and EIF3I might be a general regulator of GCH1. Our finding indicates GCH1 might have broader functions beyond tetrahydrobiopterin biosynthesis.

## Introduction

Tetrahydrobiopterin (BH4) is an essential cofactor for phenylalanine hydroxylase, tyrosine hydroxylase, tryptophan hydroxylase, and nitric oxide synthases (NOS) and alkylglycerol monooxygenase [1]. The normal BH4 level is required for the degradation of phenylalanine, the biosynthesis of catecholamine, serotonin, and the balance of nitric oxide and superoxide [2]. GTP cyclohydrolase I (GCH1) is the first and rate-limiting enzyme in the *de novo* pathway of BH4 biosynthesis [3]. Mutation of GCH1 resulted in greatly reduced BH4 levels which has been shown to cause neurological diseases such as dopamine-responsive dystonia (DRD) [4] and atypical severe phenylketonuria (PKU) [5]. Single nucleotide polymorphisms in GCH1 were associated with increased susceptibility of patients to develop neuropathic and inflammatory pain [6], [7]. Recently GCH1 has been linked to hypertension, atherosclerosis, diabetes, cardiac hypertrophy, and myocardial ischemia [2] and has become a potential therapeutic target in cardiovascular disease. Previously we have found that GCH1 confers the increased resistance to myocardial ischemia in Brown Norway rats compared to Dahl S rats [8]. Over-expression of GCH1 restores ischemic preconditioning during hyperglycemia [9], protects against acute cardiac allograft rejection [10], attenuates blood pressure progression in salt-sensitive low-renin hypertension [11], and reduces endothelial dysfunction and atherosclerosis in ApoE-knockout mice [12]. However, the understanding of molecular mechanisms of the protective functions by GCH1 remains very limited.

GCH1 is regulated by protein-protein interaction. GFRP specifically binds to GCH1 and mediates BH4 feedback inhibition and phenylalanine feed-forward stimulation of GCH1 activity [13], [14]. It was reported that the N-terminal peptide of GCH1 is the auto-inhibitory control element that contributes to bind to GFRP [15]. In endothelial cells, the phosphorylation status of GCH1 seems to impact the interaction between GCH1 and GFRP and modulate BH4 production [16]. However, the mRNAs of GFRP and GCH1 are not always co-expressed. For examples, GFRP expression is lacking in some rat tissues such as pineal and adrenal glands [3], and some species like drosophila [17]. Furthermore, in endothelial cell lines, over-expression or knockdown of GFRP did not affect GCH1 activity or BH4 production [18]. Therefore, it is important to discover other potential protein partners that are involved in GCH1 function and regulation. We and others have identified some GCH1-interacting partners in exogenous GCH1 over-expressing cell lines [19], yeast [20] and drosophila [21], however, the identities of endogenous GCH1-interacting partners in different organs remain unknown.

Accordingly, in this study we aimed at identifying the endogenous GCH1-interacting proteins in rat and characterize the interaction between GCH1 and its protein partners in different rat organs as well as the sub-cellular distribution of GCH1. We demonstrated here that GCH1 interacts with 17 proteins including GFRP in rat liver and the protein interaction profiles were quite different in brain, heart, liver and kidney except a common partner newly identified by this study, eukaryotic translation initiation factor 3 subunit I (EIF3I). In addition, GCH1 interacts with mitochondria proteins and has functional distribution in mitochondria.

## Materials and Methods

### Ethics Statement

All animal protocols were approved by the Institutional Animal Care and Use Committee of the Medical College of Wisconsin. Rats used in this study received humane care in compliance with the Guide for the Care and Use of Laboratory Animals by the National Research Council.

### Materials

Polyclonal GCH1 antibody was generously provided by Dr. Zvonimir S. Katusic [22]. Antibody against GFRP was from Abnova, antibody against tubulin beta-2A chain (TBB2A) was from Sigma. Antibodies against EIF3I, VLCAD, ALDH, and DNJA1 were from Santa Cruz Biotechnology.

### Whole Animal Vascular Perfusion

Sprague Dawley male rats (10–12 weeks) were obtained from Charles River (Wilmington, Mass).

Rats were maintained on a regular chow with unlimited access to water. Once anesthetized, the rat thoracic cavity was promptly opened and clamped to expose heart and provide drainage for blood and fluids. Then, a needle connected with a perfusion bottle containing PBS was inserted into left ventricle and a small cut was made in atrium. The perfusion pressure was maintained at ∼80 mmHg. The rat was perfused with PBS until the blood was completely cleared from the organs (∼15 min). The brain, heart, liver and kidney were rapidly excised, frozen in liquid nitrogen and stored at −80°C until use.

### Plasmid and Cell Culture

Rat GCH1 and GFRP were cloned into pcDNA5.0 vector with Flag or HA tag in the N-terminal. The plasmids were transfected into human embryonic kidney cell line (HEK) Flp-in cells (Invitrogen) by Lipofectamine 2000 (Invitrogen, Carlsbad, CA). The Flag-GCH1 stable cell line was established as previously described [23].

### Antibody Conjugation

The GCH1 antibody and normal rabbit IgG were covalently conjugated to protein A/G plus agarose by disuccinimidyl suberate (DSS) using Pierce Crosslink Immunoprecipitation Kit (Rockford, IL). The resin (20 µl) was washed with 200 µl of coupling buffer (0.01 M sodium phosphate, 0.15 M NaCl; pH 7.2) twice. The resin was then incubated with 10 µg antibody in 100 µl coupling buffer for 60 min. The antibody bound resin was washed three times with coupling buffer and crosslinked by incubating in 450 µM of DSS for 60 min at room temperature. Later elution buffer (provided with the kit, pH 2.8, containing primary amine) was added three times to remove the non-crosslinked antibody and the flow-through (the Eluates 1–3) was saved and used to verify the crosslinking. Part of the crosslinked antibody was incubated with GCH1-HEK cell lysates or rat liver homogenates overnight and eluted with the elution buffer. The flow-through of these samples, the first elution collected (Elution 1) as well as the Eluates 1 and 3 were Coomassie stained or processed for western blot analysis for GCH1 to check the efficiency of the crosslinking. A total of 300 µg GCH1 antibody was conjugated to the resin and stored in coupling buffer at 4°C.

### GCH1 Protein Complexes Purification

The perfused liver, brain, heart and kidney were homogenized in MOPS lysis buffer [23] and a total of ∼150 mg protein lysates were used in the purification. The protein lysates were pre-cleared by incubation with a mixture of protein A/G plus agarose (4 ml) and normal rabbit IgG (2 mg) for 2 hr. The supernatant was split into two to incubate either with GCH1 antibody-conjugated agarose or IgG-conjugated agarose in column with end-over-end rotation overnight at 4°C. The flow-through was saved for verification. The protein and agarose mixture were washed with 250× column volume of washing buffer (150 mM NaCl and 50 mM Tris-HCL, PH, 7.5). The IgG and GCH1 protein complexes were eluted by adding 1 ml elution buffer and incubated for 3 min. The column was eluted for 6 times and the eluate was quickly neutralized with 1 M Tris (PH 9.5). The column was then washed with 20× column volume of washing buffer twice for neutralization followed by elution with 20× column volume of elution buffer twice to clean the antibody-conjugated agarose. The column was then stored in storage buffer (50% glycerol and 0.02% sodium azide in washing buffer) at 4°C for repeat use.

### Liquid Chromatography Mass Spectrometry (LC/MS) Analysis

100 µl of the first and the second elution, respectively, were combined and precipitated with acetone. The protein pellet was dissolved in 15 µl of 400 mM ammonium bicarbonate. Five µl of the proteins solution were reduced with 5 µl of 40 mM DTT for 3 hr at 37°C followed by alkylation with 5 µl of 30 mM iodoacetic acid for 30 min at 37°C. The proteins were then digested by 1 µg trypsin (Promega, Madison, WI) and diluted in 15 µl of 400 mM ammonium bicarbonate overnight at 37°C. The digestion was quenched by adding 3 µL acetic acid. The resulting peptide mixture (4 µl) was separated by two-dimensional nano LC system (Eksigent technologies, Dublin, CA) using a Zorbax SB300-C8 trap (Agilent technologies, Santa Clara, CA) followed by reverse phase gradient onto a 0.1 mm×100 mm column (5 µm, 300 Å) packed in-house with magic C18 material (Michrom Bioresources, Auburn, CA). The separated peptide mixture was analyzed on-line with a LTQ mass spectrometer (Thermo Scientific, Bremen, Germany). In these LC/MS experiments, a full MS scan was acquired in parallel with data dependent MS/MS scans of the top five most abundant m/z peaks. MS/MS was performed with wideband activation, dynamic exclusion of 1 for 60 seconds with a list of 300 m/z and a width of ^+^/_−_ 1.5/0.5 m/z, collision energy of 35%, and noise level of 3000 NL. The peaklist-generating software is Xcalibur (2.0.5) (Thermo Scientific). The LC/MS/MS data were searched with Bioworks 3.0 (Thermo Scientific) against a rat database from Swissprot (7491 proteins reviewed, the database was current in October 2010) with its reversed sequences. Search parameters included trypsin digestion (K, R C-terminal cleavage except when next to Proline), full cleavage with 1 missed site (non-specific cleavages were not permitted), amino acid length of 6 to 100 with tolerance of 1.4 Da, dynamic modifications of methionine methylation (+14 Da) and cysteine carboxyamidomethylation (+57 Da). Mass tolerance for fragment ions is 1 amu (atomic mass unit), also could be written as ±0.5 or 500 mmu (milli mass units). Peptides were filtered by a Ranked Preliminary Score of 1 and probability of 1×e^−3^. We were able to retain high quality single scan unique peptide identifications. The peptides were independently identified; a distinct or representative protein is assigned to each peptide. The reason was to avoid “double-counting” when quantifying. We only grouped proteins for quantification. Homologous region peptides were quantified with the first protein in the database (alpha-numerically). For example, aortic actin (swissprot code ACTA) contains a peptide, QEYDESGPSIVHR, also found in cytoplasmic actin (ACTB) but was only quantified in ACTA. Results were filtered to less than 5% False Discovery Rate (FDR), defined by number of proteins identified with reversed sequences divided by the total number proteins identified minus reversed number, multiplied by 100.

### Immunoprecipitation (IP) and Western Blot Analysis

IP and western blot analyses were performed as previously reported [8]. Briefly, 1 mg protein lysates were pre-cleared with protein A resin followed by incubation with 10 µg of antibody overnight. The second day the lysates were incubated with protein A resin for 2 hr, then the precipitated proteins were washed and boiled in protein loading buffer. 10 µl of the pull-down sample or 30 µg of total protein lysates were separated by SDS/PAGE. After transferred to the nitrocellulose membrane, the membrane was incubated with primary and secondary antibodies, the bands of identity were visualized with HyGlo Quick Spray (Denville Scientific Inc. Metuchen, NJ).

### Mitochondria Isolation

Intact mitochondria was isolated from rat liver using a protocol as previously described [24]. Briefly, the perfused rat liver was minced and homogenized in IBc buffer (0.01 M Tris-MOPS, 0.01 M EGTA/Tris, 0.2 M sucrose, pH 7.4) with protease inhibitor cocktail (Sigma). The homogenate was then centrifuged at 600 g for 10 min twice at 4°C. The supernatant was transferred and centrifuged at 7,000 g for 10 min. The supernatant part was collected as the cytosol; and the pellet was washed twice and saved as mitochondria.

### BH4 Assay

BH4 was quantified on a HPLC with an electrochemical detector as previously reported [19]. Liver tissue or cells were lysed in 50 mM phosphate buffer (pH 2.6) and centrifuged (12000×g, 10 min, 4°C). The supernatants were filtered through a 10 kDa cutoff Amicon Ultra Centrifugal Filters (Millipore, Ireland) and the flow-through was analyzed for BH4 concentration by HPLC. The BH4 levels were normalized to protein concentrations.

### Data Analysis

The searched proteins were filtered using the following criteria to ensure high quality of data: 1) a high spectral count (MS/MS spectra identified as peptides for a protein) for unique proteins in the GCH1 protein complexes or the proteins detected at least twice more than the proteins in IgG complexes); 2) The proteins appeared in at least two out of five independent experiments (eliminated proteins that appeared in less than two independent experiments); and 3) some IgG proteins were removed. UniProtKB/Swiss-Prot and PubMed were used for gene ontology (GO) annotations.

Data are expressed mean±standard deviation (SD). Comparisons between 2 groups were by 2-way ANOVA, followed by post-hoc test with Duncan's Multiple Range test. A value of *P<0.05* was considered statistically significant.

## Results

### Purification of GCH1 protein complexes

As shown in Figure S1-A, there were no stained proteins in lane 2 (Eluate 3) indicating that all the unbound GCH1 antibodies were completely eluted from the agarose after crosslinking and elution. After incubation with immobilized GCH1 antibody, there was no detectable GCH1 in the flow-through from both GCH1-HEK cell lysates and liver samples by western blot analysis (Lane 3, 4 of Figure S1-B) while there was large amount of GCH1 pulled down in the Elution 1 (Lane 5 and Lane 6). These results indicate that the immobilized GCH1 antibody was very effective in pulling down endogenous GCH1 from cells and animal tissues.

**Figure 1:**
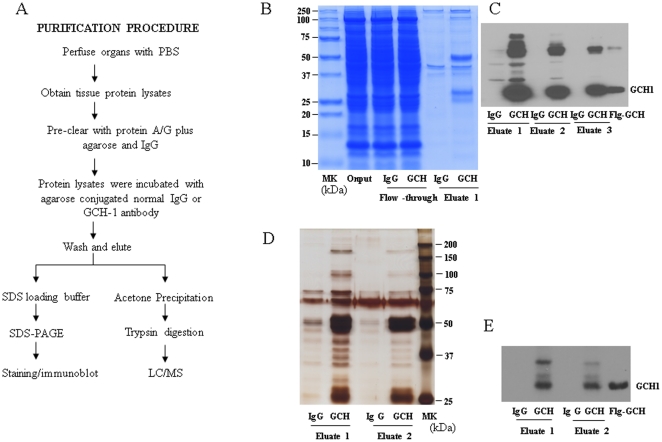
Purification of GCH1 and its interacting proteins. (A) Flow chart showing the procedure for purification and characterization of GCH1 complexes from rat organ (brain, heart, liver and kidney). (B) Protein samples from different steps of protein purification by IgG and GCH1 from rat liver were separated by SDS-PAGE, (B) stained with Gelcode Blue and (C) analyzed by Western blot analysis against GCH1 antibody. IgG and GCH1 purified samples from rat heart were (D) silver stained and (E) analyzed by Western blot analysis against GCH1. MK, molecular weight marker; Ig, IgG; GCH, GCH1; FL, flow-through; FLG, Flag tag, Flg-GCH is cell lysates from FLAG-GCH1 over-expressing HEK cells, used as positive control.

To reduce the potential contaminations of plasma proteins, we perfused the whole rat through left ventricle by PBS for 15 min. The tissue homogenate were precleared with a combination of protein A/G plus agarose and agarose conjugated IgG for 2 hr to minimize the non-specific binding to the agarose beads and the antibody. Figure 1A outlined the purification procedure. The conjugated rabbit IgG was used as a control. As there is high expression level of GCH1 in the liver, we verified the purification by running the purified samples in SDS-PAGE gel followed with coomassie staining (Figure 1B). For the purified GCH1 complexes from heart (Figure 1D), brain and kidney (data not shown in Figures), the samples were run on a SDS-PAGE followed with silver staining. As shown in Figure 1 C (liver samples) and Figure 1E (heart samples), the GCH1 protein (∼27 kDa) was able to be visualized by western blot analysis and there were strong GCH1 bands in the GCH1 antibody but not IgG pull-down complexes. The Flag-GCH1 cell lysates were used as a positive control for GCH1 as these cells are over-expressing GCH1 as we described previously [19], [23]. Compared to the IgG pull down samples, there were extra bands in GCH1 pull down eluates visualized by the staining (Figure 1B and Figure 1D), indicating that they are possible GCH1-interacting proteins.

### Identification of GCH1 interacting proteins in rat liver

**Figure 2:**
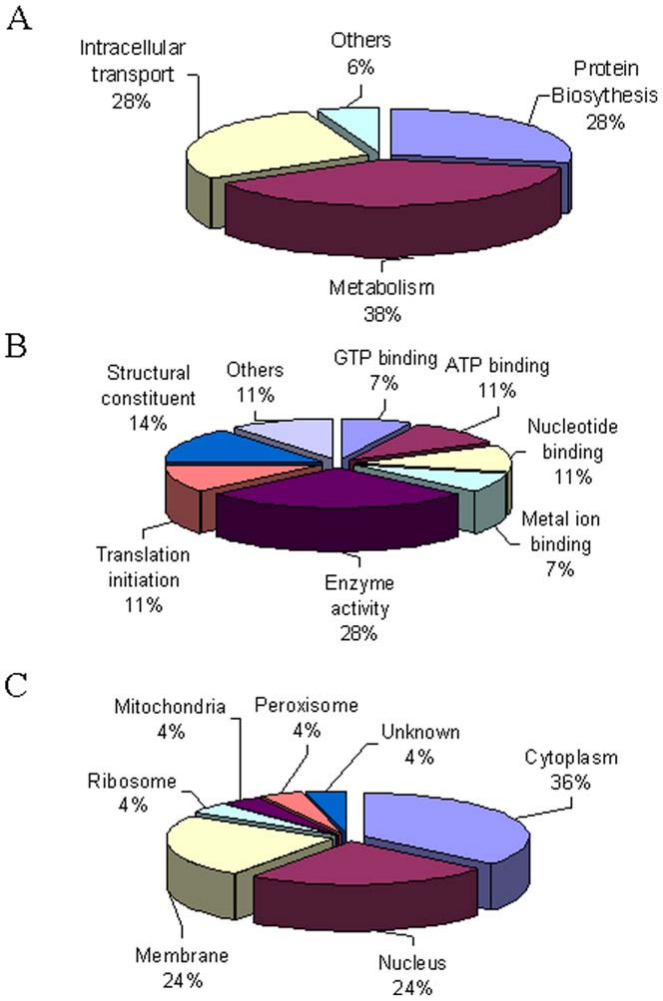
Gene ontology analysis of identified GCH1-interacting proteins in liver. The identified GCH1-interacting proteins were characterized according to their involvement in biological process (A), molecular functions (B) and cellular localization (C).

By LC/MS, we identified 564 unique peptides and 158 proteins in IgG pull-down complexes with FDR at 1.1% and 3.8% respectively. However, in the GCH1 complexes, 865 unique peptides and 289 proteins were detected with FDR of 1.6% and 4.8% (Data not shown). After filtering out the proteins that bind to IgG or have total scan count less than 2, we identified 18 unique proteins in GCH1 pull down complexes from rat liver (Table 1, Table S1). Among the 18 proteins, GCH1 was identified in all five independent (biological) repeats and had the highest scan count. Using gene ontology classification, most of the identified proteins were found related to the biological process of metabolism (38%) and protein biosynthesis (28%) (Figure 2A, Table S2). About 28% of the GCH1 interacting proteins have the molecular function of enzyme activities such as GTPase, ATPase, transferase and dehydrogenase activities; and many of these proteins bind GTP, ATP, nucleotide or metal ions (Figure 2B). Further, the GCH1-interacting proteins were diversely distributed in cytoplasm (36%), nucleus (24%), and membrane (24%) as well as mitochondria, ribosome and peroxisome (Figure 2C and Table S2). Figure S2 shows representative tandem mass spectra of tryptic peptide (DPSQIDSNEPYMK) of EIF3I, one of the identified GCH1 protein partners.

**Table 1:** Identified Proteins in GCH1 pull-down rat liver samples.

Uniprot	Gene name	Biological Process
P22288	GCH1	BH4 biosynthesis
B0BNA7	Eukaryotic translation initiation factor 3 subunit I	Protein biosynthesis
P70552	GCH1 feedback regulatory protein	Negative regulation of GCH-1 activity
P85108	Tubulin beta-2A chain	microtubule-based movement, protein polymerization
P62832	60S ribosomal protein L23	translation
P47819	Glial fibrillary acidic protein	response to wounding
P39052	Dynamin-2	receptor-mediated endocytosis
Q4KLZ6	ATP-dependent dihydroxyacetone kinase	glycerol metabolic process
Q5RK09	Eukaryotic translation initiation factor 3 subunit G	Protein biosynthesis
P04905	Glutathione S-transferase Mu 1	Conjugation of reduced glutathione to hydrophobic electrophiles
Q4G061	Eukaryotic translation initiation factor 3 subunit B	Protein biosynthesis
P45953	Very long-chain specific acyl-CoA dehydrogenase	Fatty acid metabolism, Lipid metabolism
P04904	Glutathione S-transferase alpha-3	Conjugation of reduced glutathione to hydrophobic electrophiles
P30839	Fatty aldehyde dehydrogenase	formaldehyde metabolic process, oxidation-reduction process, response to reactive oxygen species
P63036	DnaJ homolog subfamily A member 1	DNA damage response, detection of DNA damage
P16970	ATP-binding cassette sub-family D member 3	peroxisomal long-chain fatty acid import
Q6IFW5	Keratin, type I cytoskeletal 1	maintenance of corneal epithelium integrity
Q6IFV3	Keratin, type I cytoskeletal 15	intermediate filament-based process

**Figure 3:**
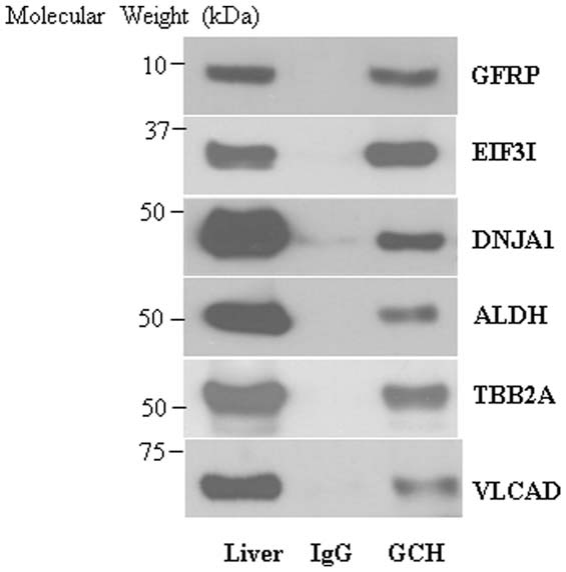
The interactions of GCH1 with its 6 protein partners identified by ESI/LC/MS were validated in rat liver by western blot analysis. The purified GCH1 complexes from rat liver were separated by SDS-PAGE and then immunoblotted with different antibodies as indicated. The blots were representatives of five independent biological repeats.

We further validated six identified proteins partners of GCH1: GFRP, EIF3I, DNJA1, ALDH, TBB2A and VLCAD by western blot analysis of pull-down complexes (Figure 3). Liver protein straight lysates were used as positive controls. All six proteins were clearly detected in GCH1 pull-down samples but not IgG pull-down samples, indicating that these proteins interact specifically with GCH1 *in vivo*. We also performed reverse IP using antibodies against these 6 proteins; unfortunately, these antibodies did not work well for IP with rat samples (Data not shown).

### The interaction of GCH1 and GFRP in different tissues

**Figure 4:**
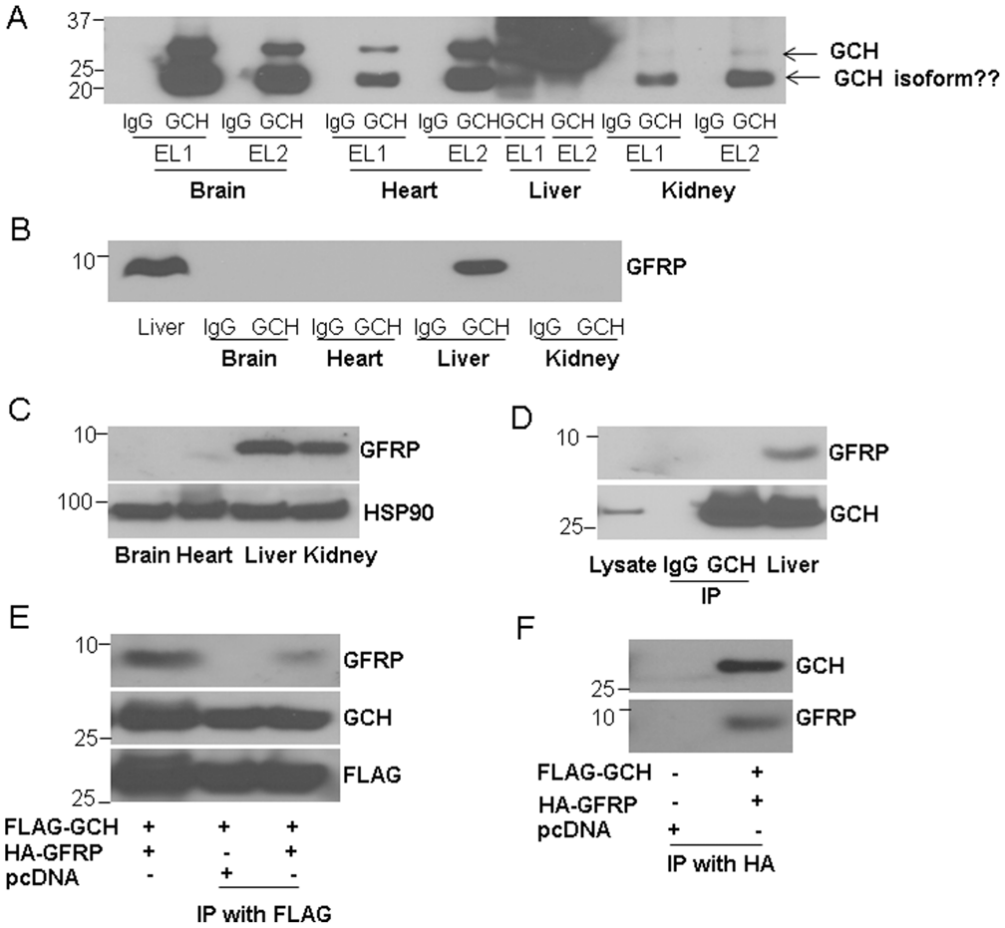
The interaction of GCH1 with GFRP in different organs. GCH1 and its interacting proteins were purified from brain, heart, liver and kidney, and analyzed by western blot against GCH1 antibody (A) and GFRP antibody (B). EL1 is the first eluate, EL2 is the second eluate. Eluates from IgG conjugated column were used as controls. Protein lysates from different organs were immunoblotted with GFRP antibody and HSP90. HSP90 was used as a loading control (C). HEK cells stably over-expressing GCH1 (GCH1-HEK) were immunoprecipitated with IgG or GCH1 antibody and immunoblotted against GFRP and GCH1. Straight cell lysate of GCH1-HEK cells (Lysate) was also loaded for comparison (the first lane). Liver homogenate was used as a positive control (D). HEK cells were transiently transfected with Flag-GCH1, HA-GFRP or pcDNA, and immunoprecipitated with Flag tag (E) or HA tag and immunoblotted with GCH1 and GFRP antibodies. In (E), cell lysates (the first lane) from HEK cells transfected with FLAG-GCH1 and HA-GFRP were used as positive controls for GFRP and GCH1 expression.

To examine whether the identified proteins in liver also interacts with GCH1 in other organs, we purified GCH1 complexes from brain, heart and kidney. IgG was used as a control. As shown in Figure 4A, GCH1 was purified from these organs by GCH1 agarose but not IgG agarose. Besides the 27 kDa band (very weak in the kidney samples), the predicted molecular weight of GCH1, there was a strong band around 20 kDa detected by western blot using GCH1 antibody in brain, heart and kidney samples, which we suspect might be a different isoform of GCH1. Interestingly, we found high levels of GFRP in the GCH1 pull-down complexes from liver samples but not from brain, heart and kidney samples (Figure 4B), although there was a high GFRP expression in kidney (Figure 4C). As GFRP is the only well-established protein that interacts with GCH1, we tested GCH1-GFRP interaction in HEK cells. When we immunoprecipitated GCH1 from the GCH1 over-expressing stable HEK cell line established by our lab [19], [23], we were unable to find GFRP in pull-down samples as well as the whole cell lysates (Figure 4D). This might be due to low expression level of GFRP in HEK cells. Liver tissue homogenate was used as a positive control for GCH1 and GFRP. Thus we further over-expressed HA-GFRP in FLAG-GCH1-HEK cells (see method). Interestingly the pull-down assay with either Flag or HA tag indeed showed the interaction between GCH1 and GFRP (Figure 4E, 4F). As the function of GFRP in regulating GCH1 activity was still controversial, we further examined whether GFRP expression affects BH4 biosynthesis. However, in the HEK cells stably expressing GCH1, we did not detect any difference in BH4 production with pcDNA or GFRP transfection (Figure S3). These results indicate that GFRP might not be the major regulator of GCH1 *in vivo* or its impact on GCH1 activity or BH4 production is tissue specific.

### The interactions of GCH1 with EIF3I, DNJA1, ALDH, TBB2A and VLCAD

**Figure 5:**
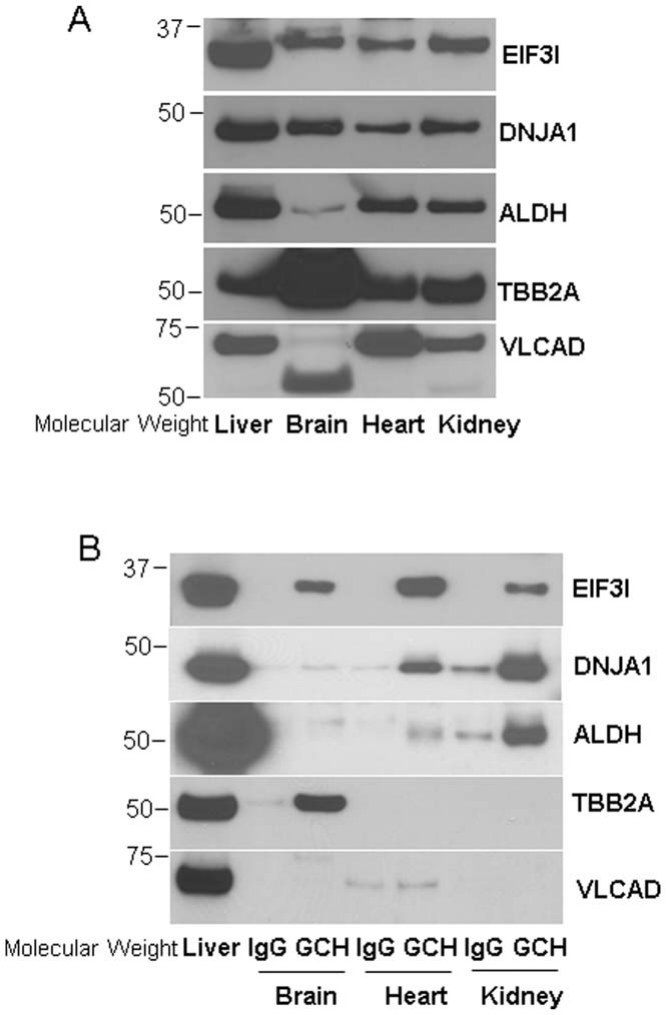
The interaction of rat liver GCH1-interacting proteins with GCH1 in brain, heart and kidney determined by western blot analysis. (A) The protein lysates from different rat organs were immunoblotted with antibodies indicated. (B) The GCH1 protein complexes purified from different organs were immunoblotted with the indicated antibodies.

Interestingly, one of the identified GCH1 protein partners in the rat liver, EIF3I, is highly expressed in all the organs we tested (liver, brain, heart, and kidney, Figure 5A). The interaction of EIF3I and GCH1 was verified by western blot analysis in all the GCH1 complexes purified from brain, heart and kidney as shown in Figure 5B. Except ALDH and VLCAD had lower expression in the brain, all other proteins are abundantly expressed in the four tested organs (Figure 5A). However, DNJA1 and ALDH only showed interaction with GCH1 in heart and kidney; TB22A bound GCH1 only in the brain; and no interaction was observed for VLCAD with GCH1 in brain, heart and kidney. These results indicate that EIF3 might be a general (ubiquitous?) interactor of GCH1 in all organs.

### The distribution of GCH1 in the mitochondria

**Figure 6:**
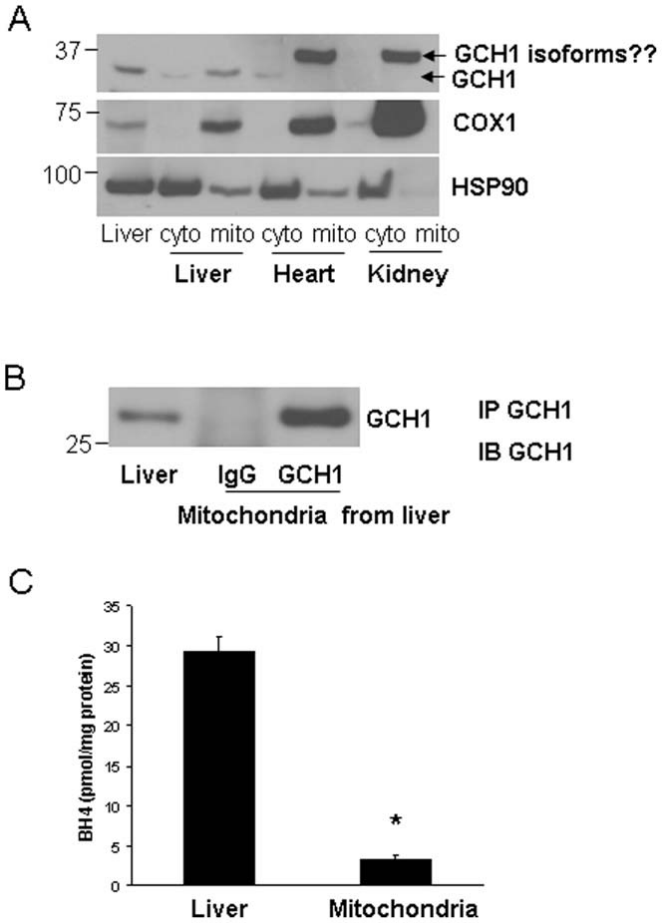
The distribution of GCH1 in mitochondria. Cytosolic and mitochondrial fractions were extracted from liver, heart and kidney and probed for GCH1, COX-1 and HSP90 by western blot analysis (A). The liver mitochondrial fraction was immunoprecipitated with IgG or GCH1 and immunoblotted with GCH1 antibody. IP, Immunoprecipitation, IB, western blot analysis (B). BH4 levels from liver and liver mitochondria were assayed by HPLC and normalized by protein concentrations (C) (N = 4). *P<0.05 vs. Liver BH4.

It was surprising to find that GCH1 interacts with VLCAD, a mitochondrial protein. The question is whether GCH1 had any distribution in the mitochondria? The mitochondrion from liver, heart and kidney were then prepared. COX-1 was used as a marker for mitochondria. As 90-kDa heat shock protein (HSP90) is mainly in the cytoplasm, thus it was used as a cytosolic marker. As shown in Figure 6A, GCH1 expressed highly in the liver mitochondrial fraction while it was almost undetectable in the mitochondrion from heart and kidney probably due to its low expression in these tissues (Figure 6A). However, there was an obvious band around 37 kDa in the mitochondrial portion of heart and kidney, which might be an unidentified GCH1 isoform. To further confirm the existence of GCH1 in the mitochondria, we pulled down GCH1 from liver mitochondrial extraction using conjugated IgG and GCH1 antibody and detected abundant GCH1 in the liver GCH1 pull-down mitochondria (Figure 6B). Additionally, we also found mitochondrial distribution of GCH1 in GCH1 HEK cells [19], [23] and hearts of GCH1 transgenic mouse (data not shown here). Furthermore, to examine the function of GCH1 in the mitochondria, we used our established methods to determine mitochondrial BH4 and we were able to detect fair amount of BH4 (3.32±1.36 pmol/mg protein) in the liver mitochondria, about 1/10th of total BH4 levels in the liver (Figure 6C).

## Discussion

The results of this study reveal several novel findings related to GCH1 regulation and function. First, we have identified 17 unique proteins interacted with GCH1 in rat liver including GFRP. Second, we have demonstrated that many GCH1 interactors interact with GCH1 in an organ specific manner. GCH1 interacts with GFRP and VLCAD in the liver, with TB22A in the liver and brain, with DNJA1 and ALDH in the liver, heart and kidney and with EIF3I in all four organs. Third, GCH1 interacts with mitochondrial proteins and itself has distribution in mitochondria. These results indicate that GCH1 might have broader functions beyond BH4 biosynthesis and the regulation of GCH1 by its interactors might be different in different organs.

It is already known that GCH1 has a number of protein partners. GCH1 interacts with GFRP in rat liver [13], [14], rabphilin-3A in PC12 cells [25], tyrosine hydroxylase in drosophila [21] and activator of HSP90 in the human brain library [20]. In our previous study, in HEK293 cell lines, we found twenty-nine proteins interacted with GCH1 when GCH1 is over-expressed in these cells [19]. However, except GFRP, none of the reported protein partners were isolated in endogenous GCH1 protein complexes from native mammalian organs. This is likely due to GCH1 has unique protein partners in different organs or cells. However, it is also possible that the exogenous over-expression may result in non-physiological interactions among proteins. To determine protein-protein interactions, pull-down assay of the endogenously expressed protein is desirable but it is also very challenging due to low abundance of the target proteins and the requirement of high-affinity antibodies. In this study, GFRP was identified consistently in liver GCH1 complexes but not IgG complexes (Table S1) indicating that our methodology works well in identifying the interacting proteins. Interestingly, GCH1 precipitated with GFRP in liver but not in kidney which also contains high GFRP levels (Figure 4B and 4C). Similarly, we have validated that except EIF3I, four other GCH1 partners identified in liver have organ-dependent interactions in other organs tested. There are several possible reasons leading to this differential protein-protein interaction. First, the post-translational modification status of GCH1 or its partners might be different in different organs. We and others have reported that GCH1 can be phosphorylated [23], [26] and ubiquitinated [27]. Phosphorylation or ubiquitination allosterically regulates proteins to associate or dissociate with other proteins. In endothelial cells, laminar shear stress causes dissociation of GCH1 and GFRP probably by increasing GCH1 phosphorylation [16]. Second, rat GCH1 might have different isoforms in different organs. Protein isoforms may have different protein-protein interaction due to conformational variability. There are at least three GCH1 isoforms identified in human liver [28] and one isoform in human monocytes [29], but up to now there is only one rat GCH1 isoform that has been reported [30]. We speculate that there might be other GCH1 splicing variants in rat as there are extra bands detected by GCH1 antibodies (Figure 1C, 4A and 6A). Also, as a 71 kDa protein, VLCAD showed a strong band at about 55 kDa in the brain but not in other tissues (Figure 5A). Third, the abundance of GCH1 or its partners might vary in different organs. The liver has the highest and the kidney has the lowest GCH1 expression level (Figure 4A). However, both the liver and kidney have comparable amount of GFRP but the endogenous GFRP is undetectable by western blot in brain, heart and HEK cell lines (Figure 4C and 4F). It is proposed that two molecules of a pentameric GFRP are associated physically with one molecule of a decameric GCH1 [14]. The mismatched GCH1 and GFRP expression might result in their distinct interaction profiles in different organs. Similarly, in the brain, ALDH has very low expression while TBB2A has much higher expression (Figure 5A). This different protein abundance might contribute to their different interactions with GCH1 in the brain.

Noticeably, EIF3I interacts with GCH1 in all four rat organs. EIF3I is a subunit of EIF3 complex which organizes a web of interactions among several EIFs required in the initiation of protein synthesis [31]. Interestingly, we have also found that other components of EIFs, EIF3G and 60S ribosomal protein L23 associate with GCH1 in the liver. Moreover, in the yeast, GCH1 is identified in the affinity purified EIF3 complexes [32]. Further studies are warranted to determine whether GCH1 participates in the regulation of translation. However, EIF3I is also an important modulator of cell signaling. EIF3I is originally identified to associate with and be phosphorylated by type II TGF-β receptor, and thus named as TGF-β receptor-interacting protein-1 (TRIP-1) [33]. EIF3I modulates the TGF-β1-stimulated wound repair [34] and gene expression response [35]. Intriguingly, over-expression of GCH1 accelerates refractory wound healing in diabetic mice [36]. As GFRP has low expression in several organs and does not interact with GCH1 in the kidney, the observation that EIF3I associates with GCH1 in all organs we tested indicates that EIF3I might work as a general regulator of GCH1 which remains to be determined.

Accumulating evidence has indicated that GCH1 might be an important regulator of mitochondrial function. The GCH1 inhibitor triggers mitochondrial depolarization in neurons [37] and mitochondrial swelling in heart [38]. Overexpression of GCH1 in the heart rescued the dysfunctional mitochondrial permeability transition pore induced by high glucose [39]. To our knowledge, our study is the first report demonstrating that GCH1 distributes in the mitochondria and interacts with fatty acid metabolism enzyme VLCAD. However, we did not find mitochondrial targeting sequence in GCH1. Thus GCH1 might translocate from cytosol into mitochondria like some nitric oxide synthase (NOS) isoforms with unclear mechanisms [40]. As BH4 is an essential cofactor for NOS isoforms, it will be noteworthy to study the regulation and function of GCH1 in relating to mitochondrial NOS. Furthermore, VLCAD has been identified to play a critical role in catabolism of long-chain fatty acids activity. The dysregulation of VLCAD is associated with production of oxidative stress and reactive oxygen species [41]. The association of VLCAD with GCH1 in mitochondria suggests that GCH1 might regulate fatty acid metabolism and exerts its protective effect to reduce oxidative stress originated from mitochondria.

The findings of this study have important implications to our understanding of GCH1 function and regulation. The identification of endogenous GCH1 differentially interacts with its partners indicates that the regulation of GCH1 is very complicated and organ-dependent. GCH1 might involve in the protein translation, fatty acid metabolism and mitochondrial function which might provide new mechanisms for its beneficial effects in cardiovascular and neurological diseases. As EIF3I has an intensive interaction with GCH1 in all organs, it might be an important and general regulator of GCH1 function.

## Supporting Information

Figure S1 **The conjugation of GCH1 antibody with agarose.** GCH1 antibody was crosslinked by using Pierce Crosslinking Kit. The resin was then washed and eluted to remove the non-crosslinked antibody. The first and third eluates (in Figure 1A, Lane 1 and 2), as well as and the flow-throughs and eluates from the conjugated antibody incubated with GCH1-HEK stable cell lines or liver samples were collected (Figure 1A and 1B, Lane 3, 4, 5, 6) and verified by Coomassie staining (Figure 1A) and by western blot analysis (Figure 1B) with GCH1 antibody (primary antibody).
(TIF)

Figure S2 **ESI/LC/MS analysis of the tryptic peptides of rat liver samples identified one of the GCH1 protein partners-EIF3I.** Representative tandem mass spectra of tryptic peptide (DPSQIDSNEPYMK) of EIF3I. Bond cleavages were indicated in the peptide sequence resulting in b/y ions.
(TIF)

Figure S3 **GFRP over-expression did not alter BH4 production in GCH1-overexpressing HEK cells.** In the HEK-GCH1 stable cell lines, pcDNA and GFRP (4 µg each) were transfected into the cells and GCH1 was induced by tetracycline for 24 hours. BH4 concentration was determined and expressed as pmol/mg protein. NS, no significant difference between the two groups (N = 3).
(TIF)

Table S1 **The spectra counts of five independent repeats from GCH1 or IgG pull-down complexes.** Ex-Experiment.
(DOCX)

Table S2 **The result of GO analysis of the identified GCH1 protein partners.**
(DOCX)
