# Supplementary figures and images for: The Protein Partners of GTP Cyclohydrolase I in Rat Organs
Source: PLoS One. 2012 Mar 27;7(3):e33991. doi: 10.1371/journal.pone.0033991 (PMC3313957; doi:10.1371/journal.pone.0033991)

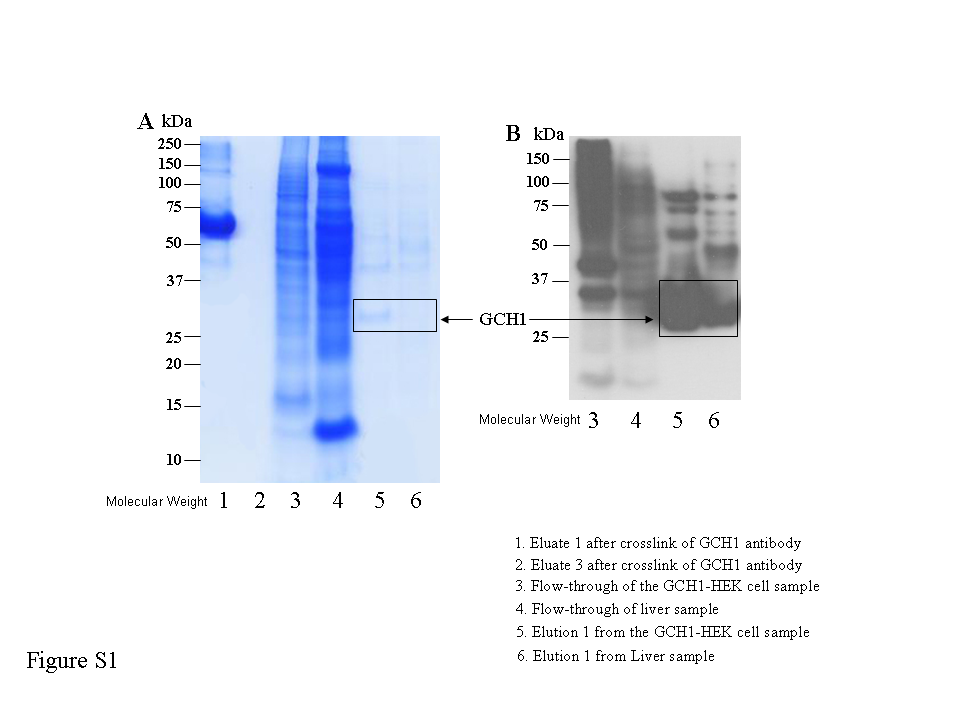

Supplement: Figure S1 — The conjugation of GCH1 antibody with agarose. GCH1 antibody was crosslinked by using Pierce Crosslinking Kit. The resin was then washed and eluted to remove the non-crosslinked antibody. The first and third eluates (in Figure 1A, Lane 1 and 2), as well as and the flow-throughs and eluates from the conjugated antibody incubated with GCH1-HEK stable cell lines or liver samples were collected (Figure 1A and 1B, Lane 3, 4, 5, 6) and verified by Coomassie staining (Figure 1A) and by western blot analysis (Figure 1B) with GCH1 antibody (primary antibody). (TIF) [file pone.0033991.s001.tif]

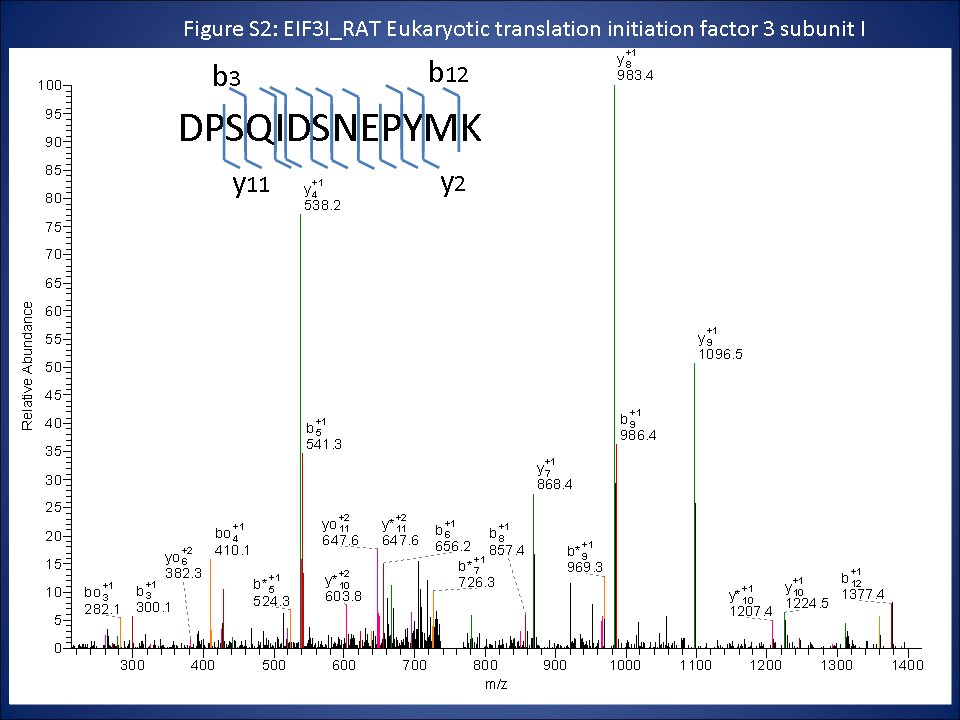

Supplement: Figure S2 — ESI/LC/MS analysis of the tryptic peptides of rat liver samples identified one of the GCH1 protein partners-EIF3I. Representative tandem mass spectra of tryptic peptide (DPSQIDSNEPYMK) of EIF3I. Bond cleavages were indicated in the peptide sequence resulting in b/y ions. (TIF) [file pone.0033991.s002.tif]

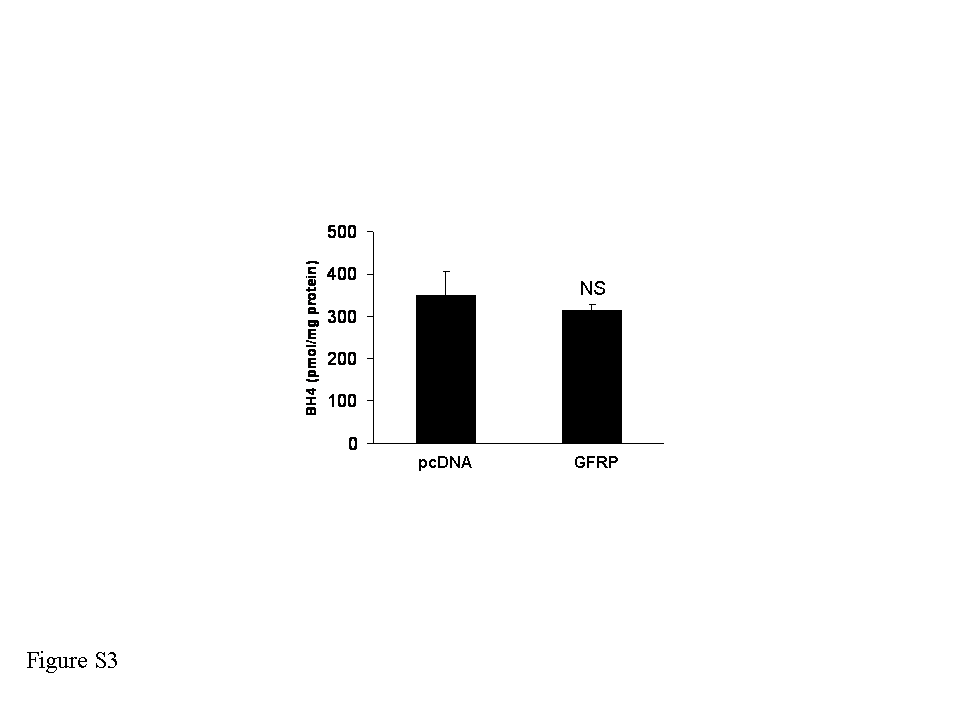

Supplement: Figure S3 — GFRP over-expression did not alter BH4 production in GCH1-overexpressing HEK cells. In the HEK-GCH1 stable cell lines, pcDNA and GFRP (4 µg each) were transfected into the cells and GCH1 was induced by tetracycline for 24 hours. BH4 concentration was determined and expressed as pmol/mg protein. NS, no significant difference between the two groups (N = 3). (TIF) [file pone.0033991.s003.tif]
