# Supplementary material for: The Protein Partners of GTP Cyclohydrolase I in Rat Organs
Source: PLoS One. 2012 Mar 27;7(3):e33991. doi: 10.1371/journal.pone.0033991 (PMC3313957; doi:10.1371/journal.pone.0033991)
Supplement: Table S1 — The spectra counts of five independent repeats from GCH1 or IgG pull-down complexes. Ex-Experiment. (DOCX) [file pone.0033991.s004.docx]

**Table S1: The spectra counts of five independent repeats from GCH1 or IgG pull-down complexes**

| **Uniprot** | **Gene name\Experiment Repeats** | GCH-ex1 | GCH-ex2 | GCH-ex3 | GCH-ex4 | GCH-ex5 | IgG-ex1 | IgG-ex2 | IgG-ex3 | IgG-ex4 | IgG-ex5 | Total SC |
| --- | --- | --- | --- | --- | --- | --- | --- | --- | --- | --- | --- | --- |
| P22288 | GCH1 | 3 | 5 | 3 | 5 | 8 |  |  |  |  |  | 24 |
| B0BNA7 | Eukaryotic translation initiation factor 3 subunit I | 2 | 4 | 1 | 2 |  |  |  |  |  |  | 9 |
| P70552 | GCH-1 feedback regulatory protein | 1 | 4 |  | 1 | 2 |  |  |  |  |  | 8 |
| P85108 | Tubulin beta-2A chain | 1 | 2 | 1 | 2 | 2 |  |  |  |  | 1 | 7 |
| P62832 | 60S ribosomal protein L23 | 2 | 1 |  | 2 | 1 |  |  |  | 1 |  | 5 |
| P47819 | Glial fibrillary acidic protein |  | 1 | 3 |  | 1 |  |  |  |  |  | 5 |
| P39052 | Dynamin-2 | 5 | 6 |  |  |  |  |  |  |  |  | 11 |
| Q4KLZ6 | ATP-dependent dihydroxyacetone kinase | 2 | 3 |  |  |  |  |  |  |  |  | 5 |
| Q5RK09 | Eukaryotic translation initiation factor 3 subunit G | 3 | 2 |  |  |  |  |  |  |  |  | 5 |
| P04905 | Glutathione S-transferase Mu 1 |  | 1 |  | 4 | 2 |  |  |  | 1 |  | 6 |
| Q4G061 | Eukaryotic translation initiation factor 3 subunit B | 1 | 3 |  |  |  |  |  |  |  |  | 4 |
| P45953 | Very long-chain specific acyl-CoA dehydrogenase |  |  |  | 2 | 1 |  |  |  |  |  | 3 |
| P04904 | Glutathione S-transferase alpha-3 |  |  |  | 2 | 1 |  |  |  |  |  | 3 |
| P30839 | Fatty aldehyde dehydrogenase | 1 | 1 |  |  |  |  |  |  |  |  | 2 |
| P63036 | DnaJ homolog subfamily A member 1 | 1 | 1 |  |  |  |  |  |  |  |  | 2 |
| P16970 | ATP-binding cassette sub-family D member 3 | 1 |  |  |  | 1 |  |  |  |  |  | 2 |
| Q6IFW5 | Keratin, type I cytoskeletal 1 |  | 1 | 1 |  |  |  |  |  |  |  | 2 |
| Q6IFV3 | Keratin, type I cytoskeletal 15 |  |  | 1 | 1 |  |  |  |  |  |  | 2 |
