# Supplementary material for: The Protein Partners of GTP Cyclohydrolase I in Rat Organs
Source: PLoS One. 2012 Mar 27;7(3):e33991. doi: 10.1371/journal.pone.0033991 (PMC3313957; doi:10.1371/journal.pone.0033991)
Supplement: Table S2 — The result of GO analysis of the identified GCH1 protein partners. (DOCX) [file pone.0033991.s005.docx]

|  | Table S2: GO Analysis of Identified Proteins in GCH1 Pull-downs | |  |  |
| --- | --- | --- | --- | --- |
| **Uniprot** | **Gene Name** | **Biological Process** | **Molecular Function** | **Cellular Component** |
| P22288 | GCH1 | BH4 biosynthesis | GTP binding,GCH1 activity, coenzyme binding, metal ion binding | Cytoplasm, nucleus |
| B0BNA7 | Eukaryotic translation initiation factor 3 subunit I | Protein biosynthesis | translation initiation factor activity | Cytoplasm |
| P70552 | GCH1 feedback regulatory protein | negative regulation of GCH1 activity | GCH1 binding ,amino acid binding | Cytoplasm, membrane Nucleus |
| P85108 | Tubulin beta-2A chain | microtubule-based movement, protein polymerization | GTP binding, GTPase activity, structural molecule activity | Cytoplasm,cytoskeleton Microtubule |
| P62832 | 60S ribosomal protein L23 | translation | structural constituent of ribosome | ribosome |
| P47819 | Glial fibrillary acidic protein | response to wounding | structural constituent of cytoskeleton | Cytoplasm, intermediate filament |
| P39052 | Dynamin-2 | receptor-mediated endocytosis | GTP binding, GTPase activity | Cytoplasm,membrane, nucleus |
| Q4KLZ6 | ATP-dependent dihydroxyacetone kinase | glycerol metabolic process | ATP binding,metal ion binding, glycerone kinase activity | unknown |
| Q5RK09 | Eukaryotic translation initiation factor 3 subunit G | Protein biosynthesis | nucleotide binding,translation initiation factor activity | Cytoplasm, nucleus |
| P04905 | Glutathione S-transferase Mu 1 | Conjugation of reduced glutathione to hydrophobic electrophiles | glutathione transferase activity,protein homodimerization activity,steroid binding | Cytoplasm |
| Q4G061 | Eukaryotic translation initiation factor 3 subunit B | Protein biosynthesis | nucleotide binding,translation initiation factor activity | Cytoplasm |
| P45953 | Very long-chain specific acyl-CoA dehydrogenase | Fatty acid metabolism, Lipid metabolism | fatty-acyl-CoA binding, flavin adenine dinucleotide binding. | Membrane, mitochondrion |
| P04904 | Glutathione S-transferase alpha-3 | Conjugation of reduced glutathione to hydrophobic electrophiles | glutathione transferase activity | cytoplasm |
| P30839 | Fatty aldehyde dehydrogenase | formaldehyde metabolic process, oxidation-reduction process, response to reactive oxygen species | 3-chloroallyl aldehyde dehydrogenase activity, aldehyde dehydrogenase activity | Endoplasmic reticulum, membrane, nucleus |
| P63036 | DnaJ homolog subfamily A member 1 | DNA damage response, detection of DNA damage | ATP binding,metal ion binding, unfolded protein binding | Membrane |
| P16970 | ATP-binding cassette sub-family D member 3 | peroxisomal long-chain fatty acid import | ATP-binding, ATPase activity | Membrane,peroxisome |
| Q6IFW5 | Keratin, type I cytoskeletal 1 | maintenance of corneal epithelium integrity | structural molecule activity | intermediate filament |
| Q6IFV3 | Keratin, type I cytoskeletal 15 | intermediate filament-based process | structural constituent of cytoskeleton | keratin filament |
